# The Activating NKG2C Receptor Is Significantly Reduced in NK Cells after Allogeneic Stem Cell Transplantation in Patients with Severe Graft-versus-Host Disease

**DOI:** 10.3390/ijms17111797

**Published:** 2016-10-27

**Authors:** Lambros Kordelas, Nina-Kristin Steckel, Peter A. Horn, Dietrich W. Beelen, Vera Rebmann

**Affiliations:** 1 Department of Bone Marrow Transplantation, University Hospital Essen, Essen 45147, Germany; Nina-Kristin.Steckel@uk-essen.de (N.-K.S.); Dietrich.Beelen@uk-essen.de (D.W.B.); 2 Institute for Transfusion Medicine, University Hospital Essen, Essen 45147, Germany; Peter.Horn@uk-essen.de (P.A.H.); vera.rebmann@uk-essen.de (V.R.)

**Keywords:** allogeneic stem cell transplantation, graft-versus-host disease, NK cells, NKG2C receptor

## Abstract

Natural killer (NK) cells play a central role in the innate immune system. In allogeneic stem cell transplantation (alloSCT), alloreactive NK cells derived by the graft are discussed to mediate the elimination of leukemic cells and dendritic cells in the patient and thereby to reduce the risk for leukemic relapses and graft-versus-host reactions. The alloreactivity of NK cells is determined by various receptors including the activating CD94/NKG2C and the inhibitory CD94/NKG2A receptors, which both recognize the non-classical human leukocyte antigen E (HLA-E). Here we analyze the contribution of these receptors to NK cell alloreactivity in 26 patients over the course of the first year after alloSCT due to acute myeloid leukemia, myelodysplastic syndrome and T cell Non-Hodgkin-Lymphoma. Our results show that NK cells expressing the activating CD94/NKG2C receptor are significantly reduced in patients after alloSCT with severe acute and chronic graft-versus-host disease (GvHD). Moreover, the ratio of CD94/NKG2C to CD94/NKG2A was reduced in patients with severe acute and chronic GvHD after receiving an HLA-mismatched graft. Collectively, these results provide evidence for the first time that CD94/NKG2C is involved in GvHD prevention.

## 1. Introduction

Allogeneic hematopoietic stem cell transplantation (alloSCT) can be an effective adoptive cellular immunotherapy for the treatment of otherwise incurable leukemia. The limitations of alloSCT are graft-versus-host disease (GvHD), infections, and leukemia relapses. These adverse clinical outcomes crucially depend on the immune reconstitution in the host. In this context it is noteworthy that natural killer (NK) cells are the first donor-derived lymphocyte population that recovers after alloSCT [1].

Although normal NK cell counts are generally observed within the first month after alloSCT regardless of the graft source, several months are required to acquire the immunophenotypic and functional characteristics of NK cells found in healthy donors. Early reconstituting NK cells exhibit a more immature phenotype expressing the inhibitory natural killer group two A (NKG2A) receptor at around 90% compared to around 50% in healthy donors [2,3]. During the NK development and peripheral maturation, the CD56dim NK cells lose NKG2A expression but up-regulate the expression of the activating NKG2C receptor, killer cell inhibitory immunoglobulin-like receptors (KIRs) and CD57 [4,5].

The NKG2A and NKG2C receptors belong to the family of the C-type lectin receptors. These receptors exist as heterodimers, commonly linked with CD94 [6,7,8]. The cognate ligand of these receptor pairs is the non-classical HLA-E molecule. HLA-E is slightly expressed ubiquitously on all healthy cells but is often up-regulated on tumor and leukemic cells [6,9]. Interestingly, for stable cell surface expression HLA-E requires a nonameric peptide repertoire comprising, preferentially, leader peptides derived from classical HLA class I A, B, and C molecules as well as non-classical human leukocyte antigen G (HLA-G) [10,11,12]. Therefore, the NKG2A/C receptors functionally monitor the biosynthesis and expression of HLA class I.

The alloreactivity of NK cells is determined by a balance of activating and inhibitory receptors including KIRs and C-type lectin-like receptor pairs [13]. In contrast to the C-type lectin-like receptor pairs CD94/NKG2A and CD94/NKG2C, the inhibitory and activating KIRs specifically recognize classical HLA class I on cell surfaces, including certain HLA-A, -B and -C molecules. Cells are susceptible to NK cell lysis either when self-HLA class I is down-regulated due to viral infection or tumorigenesis or when cells do express non-self HLA class I, e.g., in an allogeneic situation. In these cases the engagement of HLA class I with inhibitory receptors on NK cells is missing, which in turn can lead to dominant transduction of activating receptor signaling (“missing self recognition”) [14]. Along this line, donor-derived alloreactive NK cells are capable in alloSCT to eradicate residual tumor cells (the graft-versus-leukemia effect) by killing of leukemic cells. Alloreactive NK cells are also discussed to attenuate GvHD by eliminating recipient antigen-presenting cells or donor-derived alloreactive T cells [15,16].

Considering the diametrically opposed functional consequences of CD94/NKG2A and CD94/NKG2C receptor pair expression and their phenotypical changes in frequency of occurrence during NK maturation following alloSCT, it is poorly clarified whether the expression status of these receptors on donor-derived NK cells is associated with acute or chronic GvHD. To address this issue, we monitored, in a prospective study, the frequencies of inhibitory CD94/NKG2A and activating CD94/NKG2C receptor expression on NK cells in the post-alloSCT course of one year and related the results to the occurrence of severe acute or chronic GvHD in a cohort of 26 patients undergoing alloSCT. Our study revealed that the frequencies of activating receptor CD94/NKG2C on NK cells were significantly reduced in patients with severe acute or chronic GvHD during the whole observation period of one year post-alloSCT. This observation was found to be more pronounced in patients receiving an HLA-mismatched graft. Moreover, the ratio of CD94/NKG2C to NKG2A/CD94 was reduced in patients with acute or chronic GvHD after receiving an HLA-mismatched graft. In conclusion, these results provide evidence that the CD94/NKG2C receptor is associated with alloreactivity of NK cells after alloSCT, especially regarding acute or chronic GvHD prevention.

## 2. Results

### 2.1. Reduced Proportion of NK Cells Expressing the Activating CD94/NKG2C Receptor Pair in Patients with Severe Acute or Chronic GvHD

**Figure 1:**
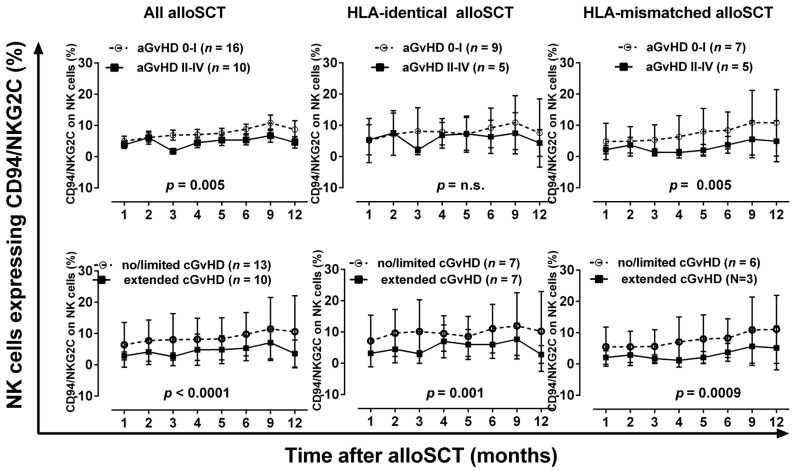
The proportions of NK cells expressing CD94/NKG2C during the course of the first year after alloSCT in relation to the occurrence of severe acute and chronic GvHD in patients receiving an HLA-matched or HLA-mismatched graft. Two-way ANOVA was used for statistical analysis.

The proportions of NK cells expressing CD94/NKG2C increase during the post-alloSCT course of one year from approximately 4% to 10% (Figure 1). Compared to 16 patients without or with only mild acute GvHD (aGvHD), the proportions of NK cells expressing the activating CD94/NKG2C receptor pair are significantly reduced in 10 patients experiencing severe aGvHD grade II–IV after alloSCT (*p* = 0.005). Moreover, the proportion of NK cells expressing CD94/NKG2C is lower in patients with extended cGvHD (*n* = 10) compared to patients without or with only limited cGvHD (*n* = 13, *p* < 0.0001). Patients after HLA-identical alloSCT do not differ in CD94/NKG2C expression profile regarding acute GvHD. However, in HLA-mismatched alloSCT patients the proportions of CD94/NKG2C-positive NK cells are significantly (*p* = 0.005) reduced in patients with severe acute GvHD (*n* = 5). For cGvHD, the proportions of CD94/NKG2A bearing NK cells are significantly reduced in patients suffering from extended cGvHD compared to patients with no or only mild cGvHD independently, whether the patients received a graft from an HLA-identical (*p* = 0.001) or HLA-mismatched donor (*p* = 0.0009). In our patient cohort, gender and age of the patients are associated neither with GvHD nor with CD94/NKG2C or CD94/NKG2A expression (data not shown).

### 2.2. Reduced Proportions of NK Cells Expressing the Inhibitory CD94/NKG2A Receptor Pair in Patients with Extended Chronic GvHD after HLA-Identical AlloSCT

**Figure 2:**
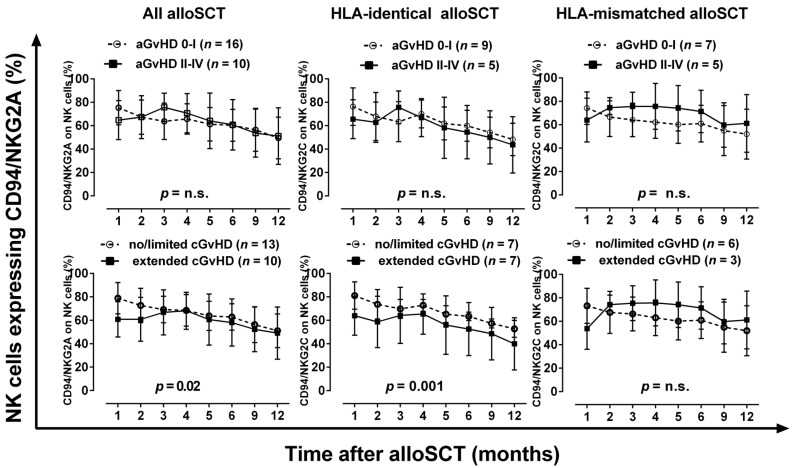
The proportions of NK cells expressing CD94/NKG2A during the course of the first year after alloSCT in relation to the occurrence of severe acute and chronic GvHD in patients receiving an HLA-matched or HLA-mismatched graft. Two-way ANOVA was used for statistical analysis.

In contrast to CD94/NKG2C, the proportions of NK cells expressing the inhibitory CD94/NKG2A receptor decrease continuously after alloSCT (Figure 2): one month after alloSCT approximately 80% of NK cells express this receptor pair, whereas only 50% of NK cells represent the CD94/NKG2A receptor pair on the cell surface after one year of alloSCT. Considering all alloSCT patients or patients receiving a HLA-matched graft, patients with extended chronic GvHD (cGvHD) display significantly (*p* = 0.02 or *p* = 0.001) reduced proportions of NK cells bearing CD94/NKG2A during the observation period of the first year post-alloSCT when compared to patients with no or only limited cGvHD. For patients with an HLA-mismatched graft, there is a trend that the proportions of CD94/NKG2A-positive NK cells increase from about 60% to 75% after two months of alloSCT in patients with acute (*n* = 5) and chronic GvHD (*n* = 3) and they maintain this level over a time period of six months, whereas in patients with no or only mild acute/chronic GvHD the proportions of this type of NK cell decrease from about 70% to 55%.

### 2.3. Reduced Ratio of CD94/NKG2C to CD94/NKG2A in Patients with Severe Acute or Chronic GvHD after HLA-Mismatched AlloSCT

**Figure 3:**
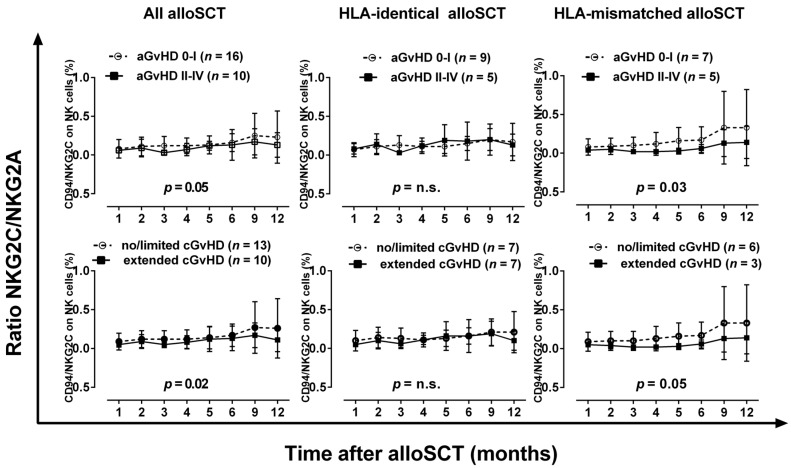
The ratio of NK cells expressing CD94/NKG2C to NK cells expressing CD94/NKGA during the course of the first year after alloSCT in relation to the occurrence of severe acute and chronic GvHD in patients receiving an HLA-matched or HLA-mismatched graft. Two-way ANOVA was used for statistical analysis.

Finally, the ratio of CD94/NKG2C to CD94/NKG2A has been calculated, as these two receptor pairs exert diametrically opposed functions. As shown in Figure 3, the ratios of CD94/NKG2C to CD94/NKG2A slightly but continuously increase during the first year after alloSCT. Among all alloSCT patients and among patients receiving an HLA-mismatched graft, patients with severe aGvHD or extended chronic GvHD (cGvHD) show a significantly lower ratio of CD94/NKG2C to CD94/NKG2A during the whole observation period post-alloSCT compared to patients with no or only mild acute or chronic GvHD. However, in patients with an HLA-matched graft the ratios of CD94/NKG2C to CD94/NKG2A do not differ among patients with and without severe acute/chronic GvHD.

## 3. Discussion

It has been proposed that alloreactive NK cells control GvHD incidence by the elimination of host-type antigen-presenting cells (APC) which prevent the priming of donor alloreactive T cells and thus GvHD [17]. Furthermore, Olson et al. demonstrated that activated donor NK cells can efficiently eliminate alloreactive donor T cells in a NKG2D-dependent manner due to the up-regulation of NKG2D ligands on the alloreactive T cell population [18]. These studies suggest that NK cells can regulate alloreactive T cells through multiple mechanisms and thus reduce GvHD incidence and GvHD-related mortality after alloSCT. Like NKG2D, the inhibitory NKG2A receptor and activating NKG2C receptor belong to the C-type lectin-like receptor family and are additional markers for NK cell development. Our prospective study on the monitoring of NKG2A and NKG2C receptor expression during the first year after alloSCT revealed (i) that, overall, the course of NKG2A and NKG2C receptor expression after alloSCT corresponds to the described phenotypical alteration of NK cell maturation [2,3,4,5]; (ii) that patients with no or mild GvHD and patients with severe GvHD display different proportions of NK cells expressing these receptors; (iii) that the association of the expression of these receptors with GvHD seems to differ with respect to the HLA-matched or HLA-mismatched allograft situation. Importantly, while NK cell alloreactivity in HLA-mismatched alloSCT can prevent GvHD, this alloreactivity is fundamental to achieving a significant antileukemic response. There are recent studies indicating an association of specific NK repertoires with less leukemia relapses after alloSCT [19,20]. Due to the very limited number of patients experiencing a relapse—One patient had a relapse within the first year after alloSCT and two additional patients relapsed after our observation period of one year—we could not establish a relationship between NKG2C expression and antileukemic response.

There is still scarce evidence regarding the recovery of the NK cell receptor subpopulations after stem cell transplantation. Picardi et al. [21] have analyzed the NK cell subpopulations with the NKG2A, NKG2C and NKG2D receptors in recipients of allogeneic SCT and autologous SCT and found a consistent up-regulation of these three NK cell receptors on CD56^+^ and CD8^+^ cells in the patients on day 30 and 90 after alloSCT. The results of this study, however, have to be viewed with caution due to a very limited patient number (allogeneic *n* = 7 and autologous *n* = 5) and the two extreme outliers among these few patients. It also appears difficult to draw a conclusion since the NK receptors were measured only at two time points (day +30 and +90). Our study describes the development of the proportion of the inhibitory CD94/NKG2A and the activating CD94/NKG2C receptor pair in more detail at eight time points over the course of one year and in 26 patients after alloSCT. Based on this monitoring, we were able to show that the NKG2A receptor continuously decreases and the NKG2C receptor increases in the first year after alloSCT, which is in agreement with the known phenotypical alteration of NK cell maturation [2,3,4,5].

Concerning GvHD, Ruggeri et al. demonstrated that alloreactive NK cells significantly improved engraftment with reduced incidence of GvHD, resulting in an overall survival benefit in patients with acute myeloid leukemia (AML) [17]. Our data point in the same direction: Patients with no or mild GvHD display a high proportion of NK cells expressing the activating receptor CD94/NKG2C during the first year after alloSCT. Additionally, we find a trend of higher proportions of CD94/NKG2C in NK cells in patients surviving the first year after alloSCT (*n* = 22) compared to four patients who died within the first year after alloSCT (Figure S1). Nevertheless, we do not find any association of CD94/NKG2C with the survival of patients in the long-term follow-up of alloSCT patients (data not shown).

Kheav et al. [22] investigated the influence of NK cell reconstitution prospectively in a cohort of 439 patients undergoing non–T-cell-depleted alloSCT and described significantly lower total and CD56dim NK cells at months three and six in patients with acute GvHD. An NK cell count above the median at month three, in contrast, was associated with a lower incidence of chronic GvHD. This study, however, has not analyzed in detail the proportions of the activating or inhibitory NK cell receptors. These studies and our results underline the importance to further investigate in detail the recovery of the NK cell subsets and receptor pairs with respect to the occurrence of GvHD and relapse. Larger patient populations are warranted to elucidate the association of NK cells for the prevention of GvHD and possibly of relapse.

HLA-E, the cognate ligand of CD94/NKG2A and of CD94/NKG2C, is currently not considered in the HLA-matching process prior to alloSCT. However, there is evidence for its influence on the outcome of alloSCT (reviewed in [23]). Danzer et al. [24] found that patients homozygous for HLA-E*01:03 enjoy a higher overall and disease-free survival and a lower transplant-related mortality (TRM) compared with patients homozygous for HLA-E*01:01. Several studies indicate a beneficial association of HLA-E*01:03 with lower risk for TRM and GvHD [25,26]. One study hypothesizes that HLA-E*01:03 might support transplant tolerance via the activation of HLA-specific regulatory T cells [23]. Since HLA-E induces NK cells expressing NKG2 receptor variants, with our results it is worthwhile to further elucidate the interplay of HLA-E and the NKG2 receptor populations on the outcome of alloSCT.

In this study we observe, overall, a reduced proportion of NK cells expressing the activating CD94/NKG2C receptor pair in patients after alloSCT with severe acute or chronic GvHD. With respect to HLA-matching, in HLA-mismatched alloSCT this association is significant for both acute and chronic GvHD, whereas in HLA-matched alloSCT there is a significant reduction of NKG2C in extended chronic GvHD only. There is evidence that donor-derived NK cells attenuate GvHD by eliminating recipient antigen-presenting cells or donor-derived alloreactive T cells [15,16]. With our results it is tempting to speculate that reduced proportions of NK cells expressing the activating CD94/NKG2C receptor pair in patients after alloSCT are associated with more severe GvHD due to their interplay with the patient’s APCs and donor-derived alloreactive T cells. The causative association between NK cell numbers, NK cell receptors and GvHD is still a matter of debate. In addition, given the limited number of patients in this study, more studies and larger patient cohorts are warranted to further elucidate the role of NK cell receptors in alloSCT. The specific NK receptor recovery and the impact on alloreactive T cells after alloSCT have to be analyzed in more detail in larger patient cohorts after alloSCT. Moreover, the role of HLA-E as the cognate ligand for CD94/NKG2A and CD94/NKG2C has to be considered in this context, too. Functional assays might help to elucidate the crosstalk of NK cell receptors and other immune cells during early reconstitution after alloSCT with respect to prevention of GvHD and of relapse.

## 4. Materials and Methods

The aim of this study is to analyze the contribution of the activating CD94/NKG2C and the inhibitory CD94/NKG2A receptor to the alloreactivity of NK cells in patients undergoing alloSCT. In total 26 patients were enrolled in the study. These patients underwent alloSCT due to acute myeloid leukemia (*n* = 20), secondary AML (*n* = 4), myelodysplastic syndrome (*n* = 1), and T cell Non-Hodgkin Lymphoma (*n* = 1). This monocentric study was planned prospectively and approved by the Ethical Board of the University Hospital of Essen. All patients signed a written consent form approved by the Ethic Committee of the University Hospital Essen to participate in this study. All patients received myeloablative conditioning. 14 patients received an HLA-identical and 12 patients an HLA-mismatched graft. The median follow-up time was about 1500 days after transplantation. GvHD diagnosis and classification was performed according to the accepted clinical standards [27]. Ten patients experienced severe acute GvHD grade II–IV and 13 patients suffered from extended chronic GvHD. One patient had acute GvHD only which did not progress to chronic GvHD; three patients had chronic GvHD without preceding acute GvHD; all the remaining patients had acute which developed into chronic GvHD. Three patients had a relapse and seven patients died after transplantation. Within the first year after alloSCT one out of three patients had a relapse and four out of seven patients died. Demographic characteristics of alloSCT patients are shown in Table 1.

Sampling, flow cytometry and statistics: Ethylenediaminetetraacetate (EDTA) plasma samples of the patients were serially procured before and one, two, three, four, five, six, nine and 12 months after transplantation and analyzed on the same day. The receptor expression profiling was performed by five-color flow cytometry using specific antibodies against CD3, CD56, NKG2A, NKG2C, and CD94. The results are given as mean plus/minus standard error of the mean. Differences in the expression profile over time were studied between patient groups by two-way analysis of variance. Statistical significance was defined as *p* ≤ 0.05.

## Figures and Tables

**Table 1:** Demographic characteristics of alloSCT patients. * Manifestation of chronic GvHD could not be evaluated for three patients due to death prior to the possible manifestation of chronic GvHD; ** Within the first year after alloSCT one out of three patients had a relapse and four out of seven patients died.

Characteristics	All	HLA-Identical AlloSCT	HLA-Mismatched AlloSCT
Number of patients	26	14	12
Median age (years (range))	51 (21–69)	51 (23–69)	47 (21–69)
Gender (female/male)	17/9	9/5	8/4
Diagnosis at alloSCT			
AML	20	11	9
sAML	4	3	1
MDS	1	0	1
T-NHL	1	0	1
Gender mismatch	13	9	4
Follow-up time after alloSCT (median days (range))	1570 (55–2004)	1570 (435–1798)	1553 (55–2004)
GvHD			
acute GvHD grade 0-I	16	9	7
acute GvHD grade II-IV	10	5	5
no/limited chronic GvHD *	13	7	6
extended chronic GvHD *	10	7	3
Relapse **	3	2	1
Alive/dead **	19/7	12/2	7/5

## Supplementary Materials

Supplementary materials can be found at www.mdpi.com/1422-0067/17/11/1797/s1.
