# Supplementary material for: The Activating NKG2C Receptor Is Significantly Reduced in NK Cells after Allogeneic Stem Cell Transplantation in Patients with Severe Graft-versus-Host Disease
Source: Int J Mol Sci. 2016 Oct 27;17(11):1797. doi: 10.3390/ijms17111797 (PMC5133798; doi:10.3390/ijms17111797)
Supplement: Supplementary file 1 [file ijms-17-01797-s001.pdf]

Supplementary Materials: The Activating NKG2C Receptor Is Significantly Reduced in NK Cells after Allogeneic Stem Cell Transplantation in Patients with Severe Graft-versus-Host Disease

Lambros Kordelas, Nina-Kristin Steckel, Peter A. Horn, Dietrich W. Beelen and Vera Rebmann

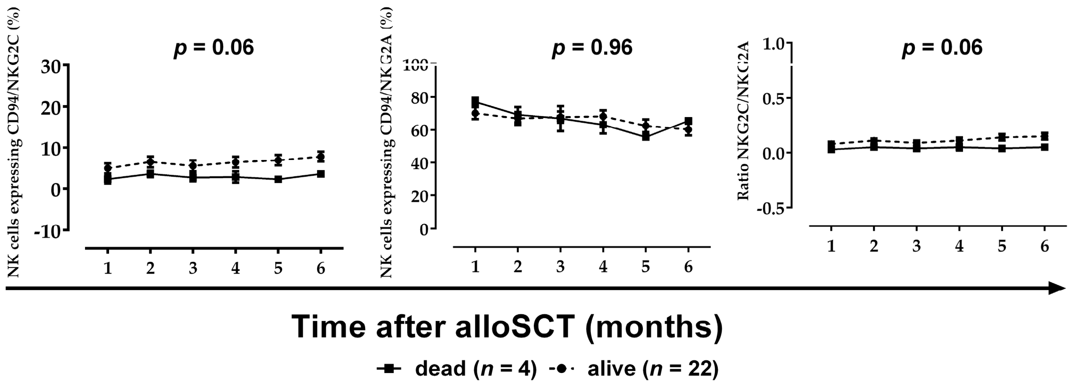

Figure S1. The proportion of NK cells expressing CD94/NKG2C, CD94/NKG2A and to the ratio NKG2C/NKG2A in relation of the survival during the first year after alloSCT. 22 patients survived the first year of alloSCT and 4 patients passed away within the first year of alloSCT. Two-way ANOVA was used for statistical analysis.
